# The Strange Case of Dr. Bruno

**Published:** 1907-02

**Authors:** M. M. Smith


### Abstracts and Selections

"THE STRANGE CASE OF DR. BRUNO." — Dr. F. E. Daniel, one of the most eloquent and pleasing writers which the South has produced, has given us in his latest work, "The Strange Case of Dr. Bruno,"* another proof of his wide culture, and deep, comprehensive sympathy with human nature, combined with scientific knowledge and literary excellence. This

*Silk cloth and gold; illustrated. By mail, $1.50. Guarantee Pub. Co., 4 W. 29th St., New York. Tobin's Book Store, Austin, Texas.

charming love story is delineated with simple, elegant force, truthful interpretation of character, a picturesque and original setting, and a loftiness of purpose plainly revealed to the close observer, who finds in the nobly expressed sentiments of Dr. Bruno a just code of moral ethics, and in the wonderful action and results of the mud wasp's sting an unusual argument for the belief in the resurrection of the body.

The action of the novel is portrayed in a manner suited to the realistic material, and is at the same time in keeping with the science which its gifted author knows so well how to interpolate. There is no crudeness of treatment, but a marked firmness and mastery detail. From the moment the reader comes in contact with the scientist, Dr. Bruno, who discovers the secret of suspended animation from the mud wasp, until the close of the career of the interesting personality, the action of the story is tempered so nicely that the incidents follow each other with discriminating correctness.

The character of Dr. Bruno is drawn with strength and firmness, and with a touch of the individualistic. His personality forces itself on the reader; his problems are real. In less experienced hands and deprived of the strength which Dr. Daniel's knowledge of science lends, the character of Bruno, whose peculiarities are accentuated by unusual incident, might show a marked tendency towards melodrama, but this result is effectively forestalled, and the tragic, human note in all its truthfulness is heard. Katie, the mother, and twenty years later Katie, the daughter, are charming, graceful figures who fulfill with ease and consistency the parts ascribed to them towards the virile, central figure, Dr. von Bruno. The main interest in the story centers about these figures, and yet the finale is not complete without the introduction of Harold Ogden, who loves Katie, and who loves twenty years later, even more madly, Katie's daughter.

The setting of this story, many of the scenes of which are laid in the quaint, historic old city of New Orleans, is picturesque, with the picturesqueness and warmth which the true Southerner loves. There are incidents of the old slave days, incidents which one ought easily believe had really occurred. Fnally there is the dramatic awakening of Bruno from a period of suspended personality in the insane asylum which recalls the tragic tales of Poe.

It cannot be doubted that the cultured author of this story held a dearer hope than that this latest child of his brain should serve only for an undiscriminating public as a "pour passer le temps." Dr. Bruno does not experiment with the magic fluid of the mud wasp with the sole intention of satiating his own curiosity, and discovering a means of suspended animation. This is the medium utilized for the expression of vital truths and noble sentiments. And high ideals are carried out also in the character of Katie, who chooses in the closing chapter rather to do violence to her feelings than to prove herself recreant to the sacred bonds of friendship.—*Dr. M. M. Smith, in Texas Medical News.*

